# Protein C Pathway in Paediatric and Neonatal Sepsis

**DOI:** 10.3389/fped.2021.562495

**Published:** 2022-02-02

**Authors:** Hassan Eliwan, Murwan Omer, Ellen McKenna, Lynne A. Kelly, Beatrice Nolan, Irene Regan, Eleanor J. Molloy

**Affiliations:** ^1^National Children's Research Centre, Dublin, Ireland; ^2^Department of Paediatrics, Trinity Translational Medicine Institute, Trinity College Dublin, Dublin, Ireland; ^3^Department of Paediatrics, Children's Health Ireland at Tallaght, Dublin, Ireland; ^4^Trinity Research in Childhood Centre, Dublin, Ireland; ^5^Department of Haematology, Children's Health Ireland at Crumlin, Dublin, Ireland; ^6^Department of Neonatology, Children's Health Ireland at Crumlin, Dublin, Ireland; ^7^Department of Paediatrics, Coombe Women's and Infant's University Hospital, Dublin, Ireland

**Keywords:** activated protein C, paediatric sepsis, neonatal sepsis, rhAPC, coagulation

## Abstract

Protein C plays a major role in the physiological regulation of coagulation pathways through inactivation of factor Va, factor VIIIa, and plasminogen activator inhibitor. Protein C is involved in the control of inflammation during sepsis, by inhibiting release of pro-inflammatory cytokines, thereby controlling neutrophil, and monocyte effects on injured tissue. Recombinant human activated protein C (rhAPC) reduced mortality in adult sepsis in earlier studies but had no significant benefit in more recent trials. Protein C levels are reduced during paediatric and neonatal sepsis, which may play a major role in the development of disseminated intravascular thrombosis, purpura fulminans, and multiorgan dysfunction. The role of protein C in paediatric sepsis requires further clinical and immunological evaluation to define the patient subgroups who may benefit from this therapy. Newer versions of rhAPC are under development with less risk of haemorrhage potentially broadening the scope of this intervention.

## Introduction

Protein C was first isolated by Johan Stenflo from bovine plasma in 1976 and named “C” because it was the third protein eluted from DEAE-Sepharose (1). It is a 62-kD, two-chain glycoprotein encoded by the PROC (protein C, inactivator of coagulation factors Va and VIIIa) gene, located on the second chromosome (2q13-q14). Protein C is an endogenous vitamin K-dependent glycoprotein that circulates in plasma as an inactive zymogen (2). The concentration of protein C in a healthy term infant ranges from 25 to 40 IU/dl and achieves adult levels after puberty (65-135 IU/dl) (3). Protein C has a half-life of 7-10 h. It is activated by thrombin bound to the cell surface glycoprotein thrombomodulin (TM) (4) to generate the anticoagulant activated protein C (APC), which has a half-life of only approximately 30 min in adult plasma. This TM-APC pathway plays an important role in maintaining homeostasis. Protein C activation by the thrombin-TM complex is enhanced by binding to the endothelial protein C receptor (EPCR) (5, 6).

Activated protein C (APC) mediates its anticoagulant function by cleaving and inactivating cofactors Factor Va (7) and FVIIIa (8). APC inactivation of FVa in plasma is dependent on protein S (9). Protein S is an anticoagulant plasma protein and a member of the protein C anticoagulant pathway, there it acts as a cofactor for APC. Protein S mediates its APC cofactor activity by enhancing the inactivation of activated coagulation FVa and FVIIIa (10).

Both protein S and procofactor FV act as cofactors to APC in inactivation of FVIIIa (11). In addition to its anticoagulant functions, APC has recently been shown to indirectly mediate protective intracellular signalling on multiple cell types. These “cytoprotective” properties result in endothelial barrier stabilisation (12), inhibition of inflammation (13–15) and apoptosis (16, 17). APC cytoprotective signalling on endothelial cells is mediated primarily by binding of APC to EPCR followed by cleavage of protease activated receptor 1 (PAR1) and PAR3 by the active site of APC (10, 12, 15–20). Alternative mechanisms for APC signalling on different cell types have also been proposed which are EPCR-independent and PAR-dependent (15) and EPCR and PAR1-independent (14).

In this narrative review the search terms: protein c, activated protein c and sepsis were used across all age-groups and included all levels of evidence in Pubmed. Adult studies were included due to the paucity of paediatric and neonatal research in this area and to explain mechanisms of action.

## Protein C Deficiency

Deficiency of protein C can be classified into two types; Type 1 describes a low level of Protein C with normal function in the circulation, which can be hereditary or acquired. In type 2, there is a normal level of protein C with abnormal function, which also can be hereditary or acquired. The hereditary form of type 1 protein C deficiency is rare genetic disorder. In heterozygote form, the activity levels of protein C are approximately 50% of normal and have a variable phenotypic expression from asymptomatic to recurrent venous thrombosis and abortion (21). The incidence of venous thromboembolism is 0.52 per 100 patient-years (22). Homozygous protein C deficiency is a rare autosomal recessive disorder with an estimated incidence of 1 in 0.5-1 million live births. Neonates may develop disseminated intravascular coagulation (DIC), purpura fulminans, vitreal vein thrombosis (absent red reflex and blindness), or perinatal ischemic stroke (23–25). The natural course is fatal unless diagnosed early and rapidly managed with replacement therapy, rhAPC. Long term management includes anticoagulant therapy with or without rhAPC supplementation. Factor V Leiden is an example of a hereditary type 2 protein C deficiency, in which there is an inability of Protein C to degrade Factor V, resulting in hypercoagulability (26). Acquired Protein C deficiency is caused by increased consumption of protein C during sepsis, hepatic synthetic dysfunction, and administration of vitamin K antagonists or as a complication of prematurity (25).

## Protein C and Inflammation

APC diminishes both the excessive coagulation and inflammatory responses in sepsis, ultimately decreasing mortality (27). In an animal model of *E. coli* infection, Taylor et al. have shown that treatment with APC protects against a lethal inflammatory response and the inhibition of protein C activation exacerbates this response (28). Protein C-deficient mice with sepsis exhibit hypotension, DIC, elevated inflammatory mediators, neutrophil adhesion to the microvascular endothelium, and loss of protective endothelial and epithelial cell barriers (29). In a double-blinded placebo-controlled trial, 16 normal adults were randomised to receive either rhAPC or normal saline, to investigate the effect of rhAPC in reducing human endotoxin–induced pulmonary inflammation. There was decreased neutrophil chemotaxis following exposure to rhAPC *in vitro*, but no difference was detected in gene expression, kinase activation, cytokine release, cell survival, or apoptosis of neutrophils (30). Inhibition of the protein C pathway increasesendothelial cell injury, cytokine elaboration and leukocyte extravasations in response to endotoxin, processes that are decreased by infusion of APC. The production of inflammatory cytokines [tumour necrosis factor (TNF)-α, Interleukin (IL)-1β, and IL-6] are inhibited by APC. It also limits the rolling of neutrophils on injured endothelium by binding selectins such as CD11b/CD18 (Mac-1), a β2 integrin involved in neutrophil signalling and cell adhesion (31).

In addition to its role in anticoagulation, APC is barrier-protective, pro-survival and has anti-inflammatory properties (32). APC is protective in ischemia-reperfusion injury by inhibition of the nucleotide-binding domain(NOD)-like receptor protein C (NLRP3) inflammasome through mammalian target of rapamycin complex-1 (mTORC1), demonstrating a potential novel way to therapeutically utilise APC (33). Neonates with sepsis have an increased risk of death if PC activity is ≤ 10%, suggesting the potential of using PC as a predictive marker of outcome in neonatal sepsis (34).

**Figure 1:**
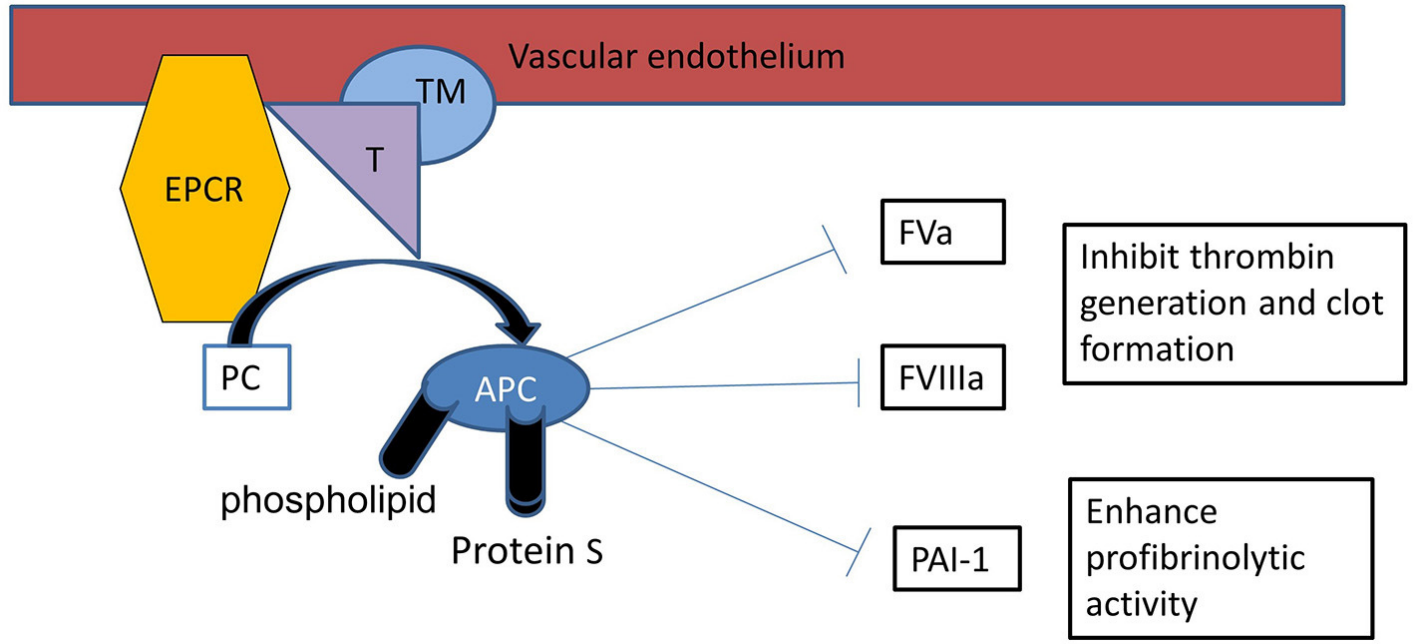
Protein C activation: Protein C binds with thrombin (T)-thrombomodulin (TM) complex on endothelial surface with it is receptor endothelial protein C receptor (EPCR), resulting in activation of protein C (APC). APC proteolytically inactivates FVa and FVIIIa, therefore APC is an important inhibitor of the clotting cascade. APC also inhibits plasminogen activator inhibitor-1 (PAI-1) enhancing profibrinolytic activity.

The Protein C anticoagulant pathway plays an important role in preventing microvascular thrombosis (35) and is initiated when thrombin binds to TM on the surface of the endothelium (36) (Figure 1). Protein C binds EPCR, augmenting protein C activation by the thrombin-TM complex more than 10-fold *in vivo* (37) and is shed from the endothelium by inflammatory mediators and thrombin. EPCR binds to activated neutrophils in a process that involves proteinase 3 and CD11b/CD18 and inhibits leukocyte extravasation. EPCR can translocate from the plasma membrane to the nucleus where it redirects gene expression, which possibly accounts for APCs ability to modulate the inflammatory responses in the endothelium (29). TNF-α and other inflammatory mediators can down-regulate EPCR and TM. APC inhibits TNF-α release from monocytes and blocks leukocyte adhesion to selectins. In addition to its classic role as a feedback inhibitor of thrombin, APC suppresses Macrophage Migratory Inhibitory Factor (MIF) and TNF-α mediated effects in the endothelial cell by down-regulating NFκB sub-units and subsequent inhibition of inflammatory cell adhesion (13). Treatment of endothelial cells with rhAPC blocks the induction of apoptosis and modulates up-regulation of the anti-apoptotic genes A1, endothelial nitric oxide synthase (eNOS) and inhibitor of apoptosis protein (38).

APC inhibits neutrophil activation in spinal cord injury and contributes to decreased ischaemic cord damage (39). Increased transcriptional expression of lung inflammatory mediators (TNF-α, IL-1β, and IL-6) in response to hyperoxia is attenuated with APC administration and associated with decreased acute lung injury (40). APC is protective in the liver and kidney after liver ischemia and reperfusion (41).

## Protein C as Treatment for Sepsis

**Figure 2:**
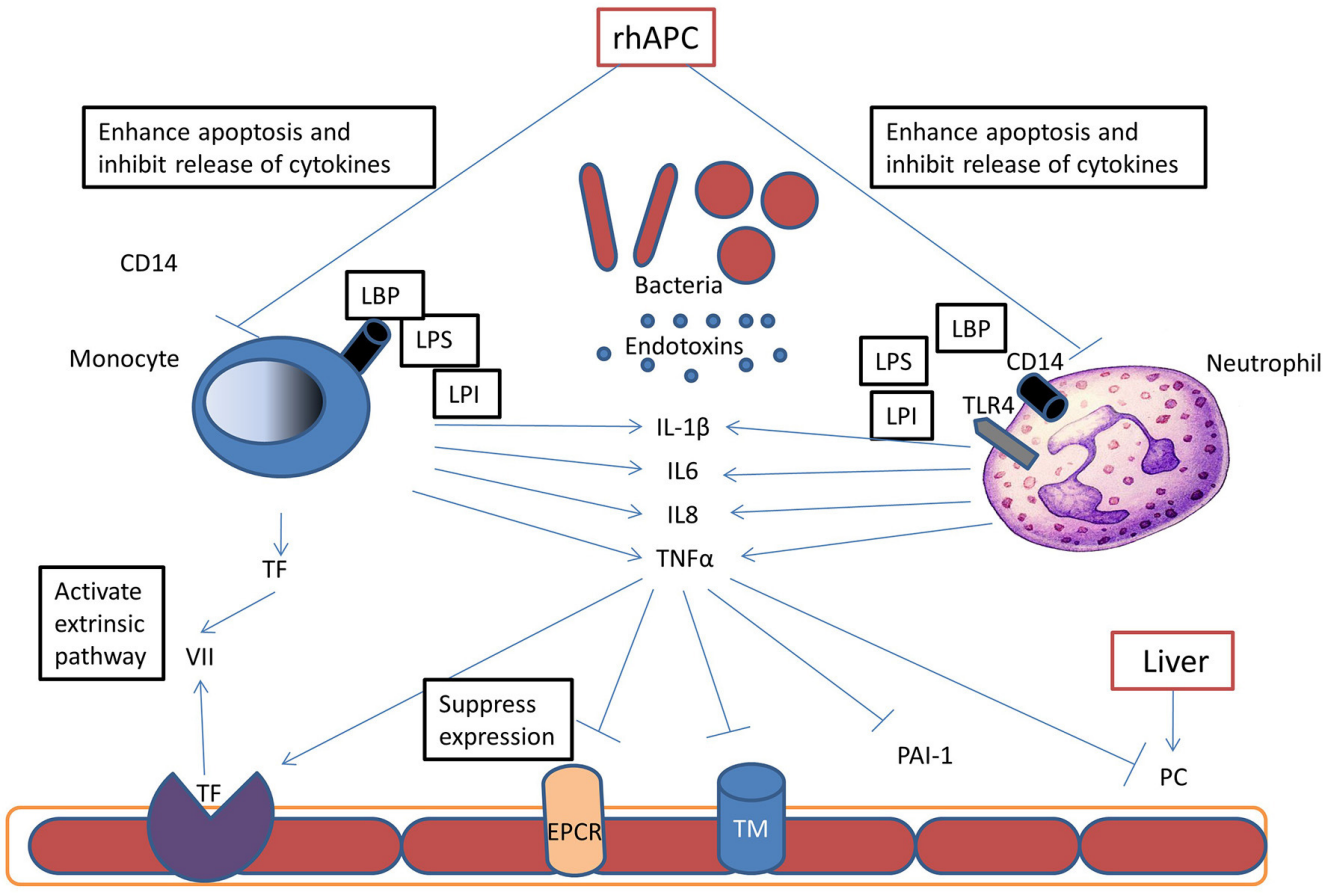
Pathophysiology of Protein C in Sepsis: during sepsis, bacteria release endotoxins (LPS, LPI, LBP), these endotoxins bind to different receptors on neutrophils and monocytes and stimulate inflammatory cytokine release. These cytokines promote thrombin generation by upregulation of tissue factor (TF) expression on monocytes and endothelium. Inflammatory cytokines also inhibit protein C synthesis in the liver and reduce thrombomodulin (TM) and endothelial protein C receptor (EPCR) expression, and thereby inhibit APC formation. Recombinant human activated protein C (rhAPC) enhances neutrophils and monocytes apoptosis and therefore inhibits cytokines release and reducing tissue damage.

Activated protein C has a major role in control of coagulation and inflammation during sepsis. Tissue injury and exposure of tissue factor in sepsis results in the activation of the coagulation cascade and thrombin generation (Figure 2). Activated phagocytic cells stimulate inflammatory cytokines mediating altered protein transcription resulting in the development of systemic inflammation. Systemic inflammation increases the synthesis of the prothrombotic proteins factor VIII, von Willebrand factor (vWF) and fibrinogen, with decreased synthesis of the regulatory proteins of the coagulation cascade-antithrombin, protein C and protein S (42, 43).

Clinical studies investigating the safety and efficacy of APC in the treatment of sepsis primarily use either recombinant human activated protein C (rhAPC or drotrecogin alpha activated) or plasma-derived protein C (Table 1). Decreased protein C levels (<50% of normal) have been associated with a poor outcome in adult severe sepsis (53). In the PROWESS (Recombinant Human Activated Protein C Worldwide Evaluation in Severe Sepsis) trial, 1690 patients diagnosed with severe sepsis were randomised in a double blind, placebo-controlled, multicentre study. They were assigned to either rhAPC or placebo. Patients on rhAPC had protein C activity less than the lower limit of normal (81%) in the 24 h before starting treatment. At day 28, 30.8% of the placebo group and 24.7% of the rhAPC group had died (*p* = 0.005) in the non-stratified analysis. There was a reduction in mortality for all individual organisms, especially *Streptococcus pneumoniae* infection (RR, 0.56; 95% CI, 0.35-0.88) (31). In the ADDRESS (the Administration of rhAPC Drotrecogin Alfa Activated in Early Stage Severe Sepsis Study Group) study, 2,640 adult patients diagnosed with severe sepsis with at least one organ dysfunction and with low risk of death were randomised in a double-blind, placebo-controlled, multicentre trial. There was no statistical difference in outcomes between placebo and treatment groups (17 vs. 18.5%; *p* = 0.34) (45). The RESPOND [Research Evaluating Serial Protein C levels in Severe Sepsis Patients on Drotrecogin alfa (activated)] study was a phase II double blind study, using endogenous protein C levels (pre-treatment, treatment and post-treatment) as a biomarker to guide the dose and duration of treatment with rhAPC. Four hundered and eighty eight patients with protein C deficiency and sepsis induced multi-organ dysfunction started treatment within 36 h of the first sepsis-induced organ dysfunction. All patients received rhAPC for 24 h and then were randomised to either a further standard 72 h of rhAPC or high dose with infusion duration dependent on their protein C level reaching the normal range. The latter strategy resulted in a greater increase in protein C levels than standard therapy. The data shows that protein C may be an effective biomarker for patients with severe sepsis and who may respond to rhAPC (54).

**Table 1:** Protein C as a treatment in sepsis.

	**Citation**	**Study group**	**Outcome**	**Key result**	**Comments**
ADULTS	Cochrane review (44)	4,911 participants: *n* = 4,434 adults; *n* = 477 paediatric patients diagnosed with sepsis.	rhAPC treatment in severe sepsis and follow up for 28 days.	rhAPC did not reduce the risk of death in adults or children (pooled RR 0.92, 95% CI 0.72-1.18; *p* = 0.50).	The effectiveness of rhAPC was not associated with the degree of sepsis severity.
	PROWESS Study (31)	Randomised double-blind, placebo- controlled, multicentre trial of rhAPC, *n* = 1,690 patients with severe sepsis.	Effect of rhAPC in the outcome of severe sepsis.	Lower risk of death in rhAPC than placebo-treated patients (*p* = 0.005).	Mortality rates was higher with *S.pneumonia* and fungal infections.
	ADDRESS study (45)	Double blind, placebo controlled multicentre randomised-controlled trial of adult patients with severe sepsis and a low risk of death, *n* = 2,613 (1,297 placebo; 1,316 rhAPC).	The effect of rhAPC at 28-days follow up for adults who had severe sepsis and a low risk of death.	No statistically significant differences between the placebo and rhAPC group (*p* = 0.34).	The rate of serious bleeding was higher in the rhAPC group, *p* = 0.02.
PEDIATRIC	RESOLVE study (46)	Double-blind, randomised, placebo-controlled, multicentre, multinational study, *n* = 477 children 0-17 years, with sepsis-induced cardiovascular and respiratory failure: assigned placebo or rhAPC.	Investigate the efficacy and safety of rhAPC. Dose used 24 microg/kg/h for 96 h.	28-day mortality (placebo 17.5%; APC 17.2%; *p* = 0.93), relative risk (RR) for 28-day mortality was 1.06 for rhAPC compared with placebo.	This study did not show any efficacy of rhAPC in children with severe sepsis. No difference in overall serious bleeding events (placebo 6.8%, rhAPC 6.7%; *p* = 0.97).
	(47)	Prospective open-label study, *n* = 8 patients, age 0.2-18.25 years with severe meningococcal disease.	Replacement therapy with rhAPC 80-120 IU/kg followed by 50 IU/kg up to six times per day as an adjunctive therapeutic regimen.	rhAPC improves coagulopathy.	No adverse effects were observed.
	(48)	*n* = 3 patients with meningococcal septicaemia.	To determine the effect of replacement therapy with rhAPC and conventional therapy.	Improvement in coagulopathy, multiple organ failure clinical features and peripheral ischemia.	No adverse events were noted.
	(49)	*n* = 30 paediatric patients with sepsis, median age 2 years.	To examine the effects of non-activated plasmid-derived protein C on amputation rate in paediatric sepsis.	A reduction in limb amputation rates compared to previous reports (16-23 vs. 50%). No bleeding events.	Plasmid-derived protein C reduces amputation in paediatric sepsis.
NEONATES	(50)	*n* = 12 patients (3 months-27 years old) diagnosed with meningococcal septic shock.	To examine the effects of protein C replacement therapy with conventional treatment in meningococcemia.	No patient deaths compared to predicted mortality score of 80 and 57%.	Protein C therapy in meningococcemia improves patient inflammatory response.
	(51)	*n* = 36 patients ( age range 3 months-72 years) with severe meningococce-mia were treated with protein C.	To investigate the efficiency of protein C replacement therapy in patients with meningococcal septicaemia.	three patients died and amputation was required in 4 patients in comparison with the predicted mortality of 50% and amputation rate of 30%.	Protein C therapy in meningococ-cal septicaemia reduces mortality and morbidity.
	(52)	*n* = 18 neonates (*n* = 12 preterm; *n* = 6 term), age 1-28 days, with severe sepsis (*n* = 6) or septic shock (*n* = 12) with coagulopathy and acquired protein C deficiency.	The effect and safety of protein C concentrate in neonates with sepsis-induced coagulopathy; Protein C, dose IV bolus of 100 IU/kg, followed by 50 IU/kg every 6 h for 72 h.	Protein C activity levels increased from 19 to 57%. During the treatment, PT reduced (*p* = 0.04), APTT (*p* = 0.02), and an increase in AT (*p* < 0.0001).	Decreased CRP (*p* = 0.005).

The EXPRESS (Early PREdiction of Severe Sepsis) study was a randomised, double blind, phase 4 equivalence design trial (*n* = 1,194) of adults with severe sepsis comparing rhAPC in combination with either heparin (low molecular weight or unfractionated heparin) or placebo. Both heparin and placebo groups had similar occurrences of severe bleeding and 28-day mortality rate (28.3 vs. 31.9%; *p* = 0.08). Patients receiving placebo had higher mortality (35.6 vs. 26.9%; *p* = 0.005) compared to a subgroup of patients in the heparin group. Significant differences were observed for bleeding events (heparin v placebo: *n* = 105 v 78; *p* = 0.049), and for ischemic stroke (heparin v placebo: *n* = 3 v 12; *p* = 0.02) (55, 56).

The Cochrane review of rhAPC in sepsis found four eligible studies with 4,911 patients (including 477 children). The risk of death was not reduced by rhAPC in adult patients at 28 days (*p* = 0.50). In two studies, there was no association between the effectiveness of APC and degree of severity of sepsis, although rhAPC was associated with significant risk of bleeding (*p* = 0.02) and they concluded that there was insufficient evidence to support the use of human rhAPC in children or adults with severe sepsis (44). Marti et al. published similar recommendations in a further Cochrane review in 2012 (44).

A multicentre, randomised, double blind, placebo-controlled study of 193 adults with severe sepsis and vasopressor-dependent hypotension receiving either an extended rhAPC infusion or placebo found no difference in mortality, organ dysfunction, and duration of hypotension (57). The extended infusion group had a lower percentage change in D-dimers (*p* < 0.001) with increase in plasma protein C activity levels. There was no difference in serious adverse events including bleeding events.

The PROWESS-SHOCK study was a placebo-controlled adult trial of rhAPC in SIRS. All-cause mortality at 28-days was the primary outcome and was not reduced by rhAPC. This prompted the withdrawal of rhAPC (*Xigris*) from the commercial market by Eli-Lilly (44, 56).

## Protein C and Paediatric Sepsis

The incidence of paediatric sepsis in the United States is 56 per 100,000 per year, and 50% of those children have an underlying disease. The annual cost is estimated at 2 billion dollars (58). Two thousand children are admitted to paediatric intensive care units in the United Kingdom annually with presumed sepsis with 20% mortality (46, 59). Protein C administration to children with febrile neutropenia significantly decreased the duration of the episode but there was no association between bacteraemia and protein C, soluble ECPR, soluble TM, and fibrin degradation products (60). In paediatric patients with septic shock, very low levels of protein C activity are associated with increased mortality (61). In children <1 year of age the protein C promoter genotype (CG-CG genotype) was associated with increased susceptibility to and development of meningococcal septicaemia with lower blood pressure and need for adrenergic support (62).

The RESOLVE study (REsearching severe Sepsis and Organ dysfunction in children: a global perspectiVE) was a randomised placebo-controlled trial, to investigate the efficacy and safety of rhAPC in children. Children aged between 38 weeks corrected gestational age to 17 years with suspected or proven infection and systemic inflammation, sepsis-induced cardiovascular and respiratory organ dysfunction within 12 h before entering the study were included. Exclusion criteria included a high risk of intracranial bleeding, end stage liver or renal disease, platelet count <30,000 or those expected to die before completion of the study. This study enrolled 477 children from 102 study sites in 18 countries who received either placebo or rhAPC (24 microgram/kg/h for 96 h). The primary endpoint for efficacy was the Composite Time to Complete Organ Failure Resolution (CTCOFR) score of three organ systems: cardiovascular, respiratory, and renal. There were no significant differences in either CTCOFR score (*p* = 0.72) or 28 day-mortality (*p* = 0.93) between patients on rhAPC compared with placebo. There were no significant differences between groups in the incidence of serious bleeding. Four out of five patients in the rhAPC group with a central nervous system bleeding event during infusion were <60 days of age and weighed <4 kg (46, 59).

The apparently conflicting results between the children in RESOLVE and adults in PROWESS with sepsis may be attributed to the baseline characteristics, trial design and statistical power or to the differences in the paediatric and adult pathophysiology of sepsis (46). In the RESOLVE study, patients with the most severe coagulation abnormalities appeared to benefit most from rhAPC and may be an important subgroup for further RCTs. This study was the largest paediatric sepsis study undertaken when published but heterogeneity of patient population and sepsis management between centres may have altered the results and larger PICU networks are required. Enrolment of few patients in many centres might have resulted in a “first patient effect,” endpoints such as cardiovascular and respiratory failure could be affected by local weaning practice. Different management in intensive care units within and between different countries might also affect endpoints reliability and numerical imbalances in some baseline characteristics because of comparatively small size. The rhAPC group had more severe disease such as more organ dysfunction, acute respiratory distress syndrome, DIC and higher paediatric risk mortality III (PRISM II). The RESOLVE study was stopped early after the second interim analysis as there was no significant difference in the primary efficacy or safety endpoints (46).

In a retrospective, multicentre study of 94 paediatric patients treated with protein C concentrate for purpura fulminans, 79.8% showed improvement, 13.8% were unchanged and 6.4% deteriorated. Skin grafts were required in 9.6% and amputation in 5.3%. The non-survivor group had lower protein C levels (*p* < 0.05), increased coagulopathy (*p* = 0.01) and longer interval to the start of protein C concentrate (*p* = 0.03) (63).

Protein C concentrate, used as an adjunctive therapy, improved coagulopathy in a prospective open-label study of eight children with severe meningococcal disease (47). In a study of Protein C concentrate administration, with daily dose adjustment to maintain plasma protein C activity range of 0.8-1.2 IU/ml, to 12 patients (3 months to 27 years old) with meningococcal septic shock, DIC, widespread purpura and skin necrosis and acquired low protein C levels, there were no deaths although the expected mortality from the Glasgow meningococcal septicaemia prognostic score and the paediatric risk of mortality score predicted a minimum mortality of 80 and 57%, respectively (50). White et al. also found a significant reduction in morbidity and mortality in patients (*n* = 36; age 0.3-72 years) with severe meningococcaemia treated with protein C (51).

A reduction in limb amputation rate, compared to previous reports (16-23 vs. 50%), was observed in a study of non-activated plasma derived protein C in 30 paediatric patients (median age 2 years) with sepsis, (49).

## Protein C and Neonatal Sepsis

Sepsis is one of the major causes of death in neonates with an incidence of 1-10 per 1,000 live births and a mortality rate of 15-50%. Protein C is decreased in healthy newborns and further diminished with delivery by uncomplicated elective caesarean section (64). Neonatal sepsis and shock are also associated with a significant reduction in plasma protein C and S levels (65). Protein C concentrate replacement therapy improved coagulopathy in 18 preterm and term neonates with severe sepsis or septic shock, coagulopathy, and acquired protein C deficiency (52).

In their Cochrane review of protein C use in neonates with severe sepsis, Kylat et al. found insufficient data to support the use of rhAPC. A cautious approach was suggested for the use of rhAPC due to the high incidence of bleeding with its use, as severe sepsis in preterm infants is commonly associated with bleeding problems and intraventricular haemorrhage. The use of rhAPC was not recommended outside the confines of randomised controlled trials (66). Infants with neonatal encephalopathy (NE) have an altered inflammatory response in terms of neutrophil reactive oxygen species (ROS) and Toll-like receptor (TLR)-4 expression. This response was reduced following treatment with APC, demonstrating the potential use of APC as an adjuvant therapy in NE (67).

## Protein C as Biomarker in Sepsis

Protein C level may be a good biomarker in sepsis. In the RESPOND study, protein C levels were used to modify therapy with increased dose and duration of rhAPC in severe deficiency (54). In a multicentre study of adult patients with severe sepsis, at baseline and 44 h, lower protein C levels were associated with increased 30 day-mortality (*p* = 0.19 and *p* = 0.04 respectively), increased incidence of shock (*p* = 0.01; *p* < 0.001) and a reduction in the number of ventilator-free days (*p* = 0.02; *p* = 0.03) (53).

In 187 patients (term newborn to <18 years old) in the ENHANCE (drotrecogin alfa (activated) in children with severe sepsis) study, there was a significant association between increased 28-day mortality and decreased protein C level following rhAPC administration (*p* < 0.001). The change in protein C level from baseline was predictive of survival (68). In a paediatric study of protein C concentrate in purpura fulminans, non survivors had lower protein C levels (*p* < 0.05), increased coagulopathy (*p* = 0.01) and an increased interval between admission and commencing protein C concentrate (*p* = 0.03) (47). In 73 patients (1 month-15 years) with septic shock, there was significantly lower protein C activity in non-survivors (*p* = 0.002) (61). Protein C levels did not differ between paediatric febrile neutropenia with and without bacteraemia in 73 paediatric oncology patients in a prospective cohort study (69).

In 40 neonates (1-28 days of age) with low birth weight, severe sepsis and organ dysfunction, plasma protein C activity levels were lower in non-survivors vs. survivors (*p* < 0.001). The positive predictive value of the protein C level for mortality was 90.9%. Each 1% rise in protein C activity levels decreased the hazard of dying by 5% Infants with congenital anomalies, birth asphyxia or blood products received before protein C assay were excluded (70).

In a prospective observational study of protein C and antithrombin III levels in neonates with late onset sepsis, mean birth weight 1.86 (range 0.7-4.2) kg, gestational age 32.7 (range 25-41) weeks, age 14.5 (8–172) days, there were statistical differences in AT III (*p* = 0.003) and protein C activity levels (*p* = 0.002) between survivors and non-survivors. Low protein C activity levels also significantly correlated with the risk of death in neonates with sepsis (*p* = 0.0016) (71). El Beshlawy et al. studied the effect of sepsis on protein C, S and ATIII levels in term neonates (30 septic and 30 normal controls). Blood samples were taken 72-96 h after birth and the physiological inhibition system including protein C, S and ATIII was markedly decreased in all septic neonates, compared to controls (*p* < 0.001). DIC developed and death occurred in 33.3% of cases; necrotizing enterocolitis in 40% of cases; rectal bleeding in 33.3%; haematuria in 20% of cases; haematemesis in 26.7% of cases; intracranial haemorrhage in 23.3% of cases, and convulsions in 23.3% (Table 2) (53).

**Table 2:** Protein C as a biomarker of sepsis.

	**Citation**	**Study group**	**Outcome**	**Key result**	**Comments**
ADULTS	ENHANCE study (68)	Single-arm, open-label, multicentre study. *n* = 188 patients (term newborn to <18 yrs old) with severe sepsis were given rhAPC 24 μg/kg/hr for 96 h.	Protein C levels, safety information and 4-day/ 28-day all-cause mortality were measured.	57.6% of patients were severely deficient in protein C (<40% of normal). Association between increased 28-day mortality and decreased protein C at the end of infusion was statistically significant.	27.7% of patients had bleeding events. 3.2% of patients had serious bleeding events. Conclusion was not possible without a placebo control.
	(69)	Prospective cohort study. *n* = 73 paediatric oncology patients admitted with febrile neutrophilia, (10 bacteremic).	Protein C levels and bacteremia in children with febrile neutropenia were assessed.	Median level of protein C in bacteremia was 0.64 U/ml and without bacteremia was 0.73 U/ml.	Protein C levels do not differentiate between paediatric febrile neutropenia with and without bacteremia.
	RESPOND study. (54)	Phase II double blind study, protein C was measured before, during and after treatment with rhAPC in 488 patients with sepsis and multiorgan dysfunction.	Evaluation of endogenous protein C levels as a biomarker to guide the dose and duration of treatment with rhAPC.	Serial measurement of protein C level identified patients with severe sepsis who would benefit from rhAPC.	Authors concluded that phase 3 study is required to evaluate if the increasing protein C level is associated with mortality reduction.
	(61)	67 blood samples collected from paediatric patients diagnosed with septic shock.	To determine protein C and D-Dimer level in survivor and non-survivor groups.	Protein C activity were lower in non-survivor compared to survivor patients (*p* = 0.002). There was no correlation between plasma PC activity and D-Dimer levels (*p* = 0.6).	Low protein C activity in paediatric patients with septic shock may be associated with an increased risk of death.
	(63)	National, retrospective, multi-centre study, *n* = 94 paediatric patients, treated with rhAPC for purpura fulminans.	To evaluate the prognostic significance of protein C plasma levels.	Non-survivor group had lower protein C plasma levels (*p* < 0.05).	Non-survivors had higher prevalence of coagulopathy at admission (*p* < 0.01).
	(70)	n = 40 neonates with low birth weight diagnosed with sepsis and organ dysfunction.	To evaluate protein C level in low birth neonates with sepsis.	APC levels in non-survivors were lower than in survivors, *P* < 0.001.	The positive predictive value of APC level for mortality was 90.9%.
	(71)	Prospective, observational study of 150 neonates with suspected late-onset sepsis.	To determine the prognostic value of plasma AT III (and PC functional levels)	ATIII and PC were significantly lower in neonates with sepsis. ATIII and PC were lowest in non-survivors compared with survivor (ATIII *P* = 0.003; PC *P* = 0.00002).	The correlation between plasma PC functional level and risk of death was statistically significant (*P* = 0.0016).
	(43)	A clinical study involving 30 septic neonates and 30 healthy neonates.	To study the effect of sepsis on the level of protein C, S and ATIII.	There was a marked decrease of protein C, S and ATIII levels in sepsis patients compared to control group (*p* < 0.001).	Protein C is useful biomarker in severe sepsis.

## Activated Protein C: Side Effects

Treatment with rhAPC has been associated with an increased risk of bleeding. The PROWESS study reported serious bleeding events in 3.5% of rhAPC treated patients, compared with 2% of those on placebo (*p* = 0.06) (31). Patients with activated internal bleeding, haemorrhagic stroke within 3 months, intracranial/intraspinal surgery or severe head trauma within 2 months, trauma with increased risk of life-threatening bleeding, presence of an epidural catheter and intracranial neoplasm or mass lesion or evidence of cerebral herniation were excluded (31). In the ADDRESS study, the rate of serious bleeding was greater in the rhAPC group than placebo group both during infusion (2.4 vs. 1.2%, *p* = 0.02) and the 28-day study period (3.9 vs. 2.2%, *p* = 0.01). Four (0.3%) patients had CNS bleeding during rhAPC infusion vs. 3 (0.2%) in the placebo group (45). In the ENHANCE study, the risk of bleeding related to administration of rhAPC was 3.2% (68). Fumagalli et al. demonstrated that adverse events associated with rhAPC occur with a frequency of 5% or greater including ecchymosis (23.0%), headache (30.9%), and pain (5.8%) primarily at the site of venepuncture. rhAPC antibody formation was uncommon (<1%) after a single course of therapy (72). In the EVAA dose finding study, rhAPC was safe and well tolerated by adult patients with sepsis (27).

A mutant APC generated with three alanine mutations (3K3A-APC) has been created with potent neuroprotective properties and reduced anticoagulant activity (73). 3K3A-APC crosses the intact blood-brain barrier through EPCR-mediated transport and may have direct action on neurons contributing to the observed neuroprotection *in vivo*. The creation of mutant APC with less anticoagulant activity may increase the utility of APC in sepsis especially in populations vulnerable to haemorrhage.

There are also many alternative compounds to rhAPC that are currently in Phase III clinical trials which include TFPI, TNF-antibody fragment, antithrombin III and platelet-activating factor acetylhydrolase (74), although TFI and antithrombin have been ineffective against severe sepsis and septic shock (75, 76).

## Other Inflammatory Conditions

### Acute Lung Injury

A recent double-blind placebo-controlled study in a human model of endotoxin-induced pulmonary inflammation, inhalation of activated protein C significantly reduced lung inflammation and leukocyte infiltration (30). In awake sheep, rhAPC alleviates endotoxin-induced lung injury as characterised by improved oxygenation and reversal of pulmonary hemodynamic and volumetric changes, as well as by reversal of pulmonary hemodynamic and volumetric changes (77). Inhaled protein C may attenuate acute pulmonary inflammation and inhaled APC improved oxygenation and lung aeration in a sheep model of endotoxin-induced acute lung injury but did not alter haemodynamics. Further research is required on the effectiveness of the inhaled route (78).

Coronavirus disease 2019 (COVID-19) has quickly spread across the world and caused a significant burden on healthcare. Patients hospitalised with COVID-19 may present with sepsis, pneumonia and acute respiratory disease syndrome (ARDS) (79). It has been established that COVID-19 patients with pneumonia have defects in their thrombin coagulation system, causing microthrombosis and multi-organ failure (79, 80). PAR-1 antagonists have been shown to be effective in a mouse model of pneumonia, and Jose and Manuel hypothesised that targeting it, may be effective in the management of thromboembolism (81, 82). Therefore, the use of therapies targeting key components of the thrombin coagulation system have the potential to control microthrombosis in the fight against COVID-19 (82).

### Brain Injury

Activated protein C (APC) protects neurons and endothelium from ischemic injury. Early administration of wild-type APC to mice with traumatic brain injury results in decreased lesion volume and improved functional outcome (83), while late administration of an APC variant with reduced anticoagulant function has been shown to be more neuroprotective than wild type APC, and has shown to significantly improve functional recovery and with no increased risk of bleeding (84). Activated drotrecogin-alfa, a form of human recombinant APC, is currently being studied in patients with ischemic stroke. Non-anticoagulant 3K3A-APC exhibits greater neuroprotective efficacy with no risk for bleeding compared with drotrecogin-alfa activated (73). APC attenuates the lipopolysaccharide (LPS)-induced protein expression of inflammatory cytokines, TNF-α and IL-6in neonatal rat brains (85). A single intraperitoneal injection of rhAPC to neonatal rats had similar protective and anti-inflammatory effects against maternally administered LPS. APC may therefore provide protection against an endotoxin-evoked inflammatory response and white matter injury in the developing rat brain.

Independent of its anticoagulant activity, APC acts directly on cells and can alter gene expression profiles, inhibit apoptosis, and down-regulate inflammation mediating neuroprotection. These effects require protease-activated receptor-1 and the endothelial protein C receptor. In an *in vitro* model involving hypoxia-induced apoptosis of human brain endothelial cells, protease-activated receptor-1 and endothelial protein C receptor is required for APC to exert its anti-apoptotic effects. In these cells, APC attenuates the hypoxia-induced increases in p53 messenger RNA and protein, reduces pro-apoptotic Bax, and increases anti-apoptotic Bcl-2, and therefore inhibits mitochondrial-dependent apoptosis. Murine ischemic stroke model studies have provided *in vivo* evidence for the physiologic roles of protease-activated receptor-1 and endothelial protein C receptor in the neuroprotective activities of APC (86). The protein C pathway is also thought to be critically involved in chronic inflammatory disorders such as inflammatory bowel disease and renal inflammation (87, 88).

### Other Disease Models

APC administration has been shown to be beneficial in neurological conditions besides stroke, slowing disease progression and prolonging survival in a mouse model of amyotrophic lateral sclerosis (ALS) (89) and demonstrating therapeutic efficacy in a mouse multiple sclerosis model (90). APC also attenuates glomerular and endothelial injury in diabetic mice (91) and has been shown to be beneficial in a mouse model of inflammatory bowel disease (92).

## Future Directions for Activated Protein C

The “holy grail” would be to develop a recombinant human APC which has improved ability to mediate protective signalling, but which has minimal potential to cause bleeding. “Second generation” recombinant APC variants have been designed, which have similar signalling properties to wild type APC but reduced anticoagulant properties (93–95). Mosnier et al. (93) generated a recombinant APC variant with impaired FVa substrate recogition due to mutation of its FVa-binding exosite. Harmon et al. have described an APC mutant with attenuated protein S cofactor function (94). Another group engineered an APC variant in which a disulfide bond was introduced which virtually ablated its anticoagulant function (95). However, until recently no means of enhancing APC protective signalling has been reported.

Ní Ainle et al. characterised novel recombinant APC variants with greatly enhanced cytoprotective signalling function but limited anticoagulant activity (96). One of the recombinant variants described in this study, APC-*L38D/N329Q*, represents the first example of an APC molecule with enhanced cytoprotective function but attenuated anticoagulant function. These novel recombinant molecules are predicted to have enhanced therapeutic potential compared to existing recombinant APC preparations when used in the treatment of systemic inflammatory conditions. A recombinant human form of endogenous activated protein C (drotrecogin alfa) was recently approved by the Food and Drug Administration for adults with severe sepsis who have a high risk of death. It possesses anticoagulant, profibrinolytic, and anti-inflammatory properties (74).

In the setting of cancer, staging systems are used to stratify patients based on risk and potential benefit from treatments (97). This approach will be key for the management of sepsis as patients are highly heterogenous in terms of the cause of sepsis, disease severity and associated co-morbidities (97). Therefore, a lack of recognition of septic patient heterogeneity in clinical trials may mask therapeutic effects of a therapy. For instance, the PROWESS trial showed reduced mortality in severe sepsis patients and the clinical trials preceding this did not, but these patients presented with a low risk of mortality to begin with. APC causes a reduction in reactive oxygen species (ROS) production after induction with LPS in adults but no difference in paediatric intensive care unit (PICU) patients. These differences in immune response to APC could explain conflicting results between the RESOLVE and PROWESS trials (98). Future studies must shift their focus to developing validated patient stratification methods if there is any hope for management of sepsis (97). This must take into account the above-mentioned factors such as the severity of disease, patient age, the initial source of infection, varying degrees of SOFA score, APC levels at baseline and the timing of treatment. The heterogeneity of sepsis is also influenced within trial centres and their sepsis criteria or definitions (99), all of these factors combined will provide a more precise clinical understanding of sepsis and outcomes. The definitions and clinical criteria made by the Sepsis-3 group provides a guideline to the progress of this (100). Similarly, these challenges are described by McGovern et al., within a neonatal population (101).

## Conclusion

Protein C levels have been used as biomarker in paediatric and adult sepsis and low protein C levels predict the risk of morbidity and mortality. Replacement therapy with rhAPC in adult sepsis reduced mortality in early studies but these results could not be replicated in more recent trials. In addition, the RESOLVE study in paediatric sepsis did not show a similar improvement in morbidity and mortality. Although activated protein C has promise for sepsis management, the development of newer preparations with decreased coagulability while retaining anti-inflammatory properties are required. Immune profiling of neonates and children treated in randomised controlled trials of rhAPC will also be important to evaluate the individualised immune responses at each stage of sepsis from proinflammatory to the compensatory anti-inflammatory response to allow treatment optimisation with medications such as rhAPC. In addition, studies with long-term outcomes beyond 28 days are vital to evaluate the effect on neurodevelopmental outcome. Therefore, large clinical trials are required to elucidate the effectiveness of rhAPC in reducing mortality and adverse outcomes in children with severe sepsis.

## Author Contributions

HE conducted the research and wrote the manuscript. EJM supervised the work and co-wrote the manuscript. MO, EM, BN, and IR assisted in writing and revision of the manuscript. All authors contributed to the article and approved the submitted version.

## Funding

This work was supported by National Children's Research Centre, Crumlin, Dublin, Ireland.

## Conflict of Interest

The authors declare that the research was conducted in the absence of any commercial or financial relationships that could be construed as a potential conflict of interest.

## Publisher's Note

All claims expressed in this article are solely those of the authors and do not necessarily represent those of their affiliated organizations, or those of the publisher, the editors and the reviewers. Any product that may be evaluated in this article, or claim that may be made by its manufacturer, is not guaranteed or endorsed by the publisher.
